# Supplementary material for: Genome-Scale Investigation of GARP Family Genes Reveals Their Pivotal Roles in Nutrient Stress Resistance in Allotetraploid Rapeseed
Source: Int J Mol Sci. 2022 Nov 21;23(22):14484. doi: 10.3390/ijms232214484 (PMC9698747; doi:10.3390/ijms232214484)
Supplement: Supplementary file 1 [file ijms-23-14484-s001.zip › ijms-2003350-supplementary-updated/Supplementary Figures.pdf]

Supplementary Figures

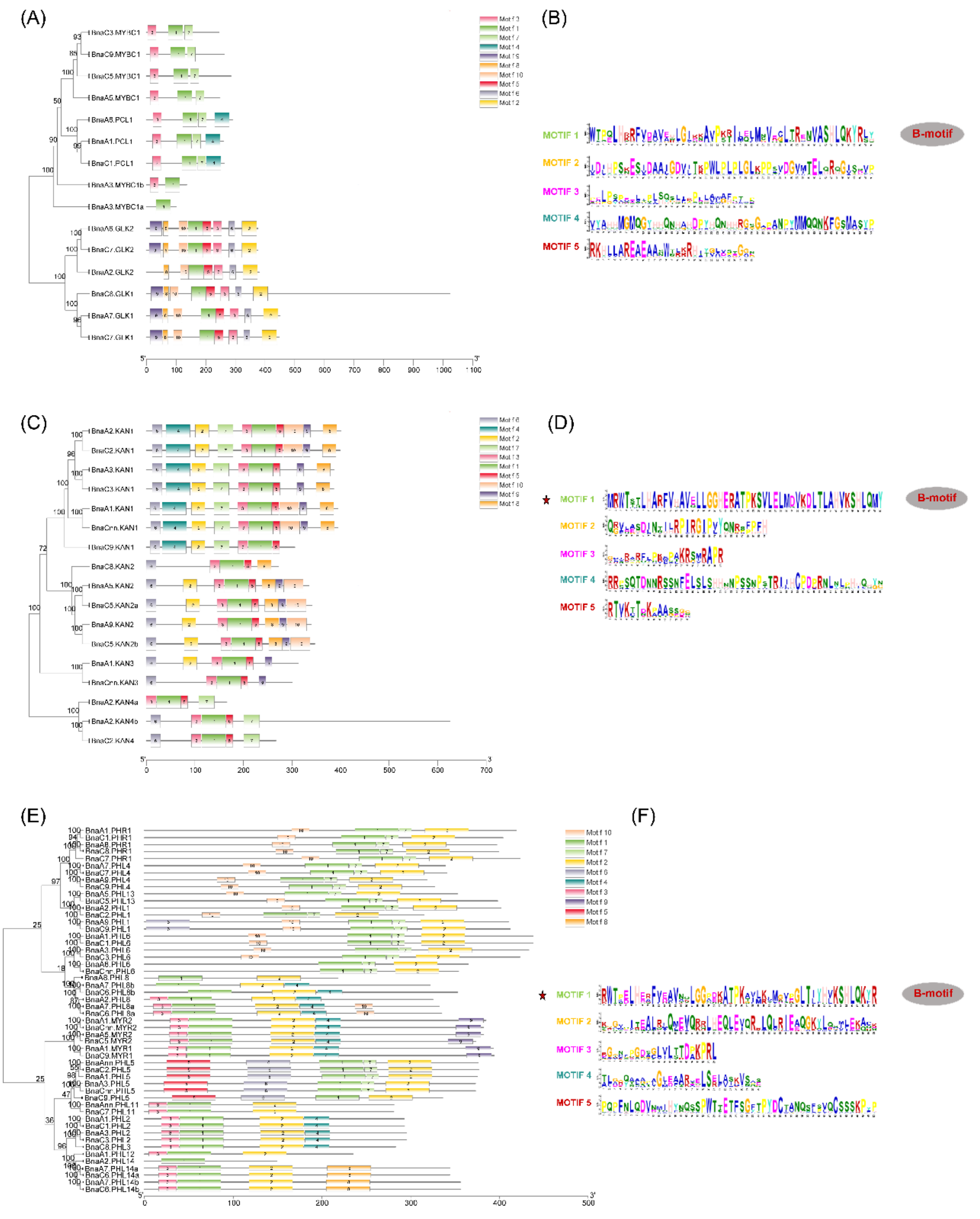

Figure S1 Identification and characterization of the conserved motifs in the GARP proteins in *B. napus*. (A) Molecular identification of BnaGLKs. (B) The sequence characterization of BnaGLKs. (C) Molecular identification of BnaKANs. (D) The sequence characterization of BnaKANs. (E) Molecular identification of BnaPHLs. (F) The sequence characterization of BnaPHLs. In A, C and E, the boxes with different colors indicate different conserved motifs (motifs 1–10), and black lines represent the GARP protein regions without detected motifs. In

B, D and E, the larger the fonts, the more conserved the motifs. Among them, the tagged motifs were identified as the B-motifs.

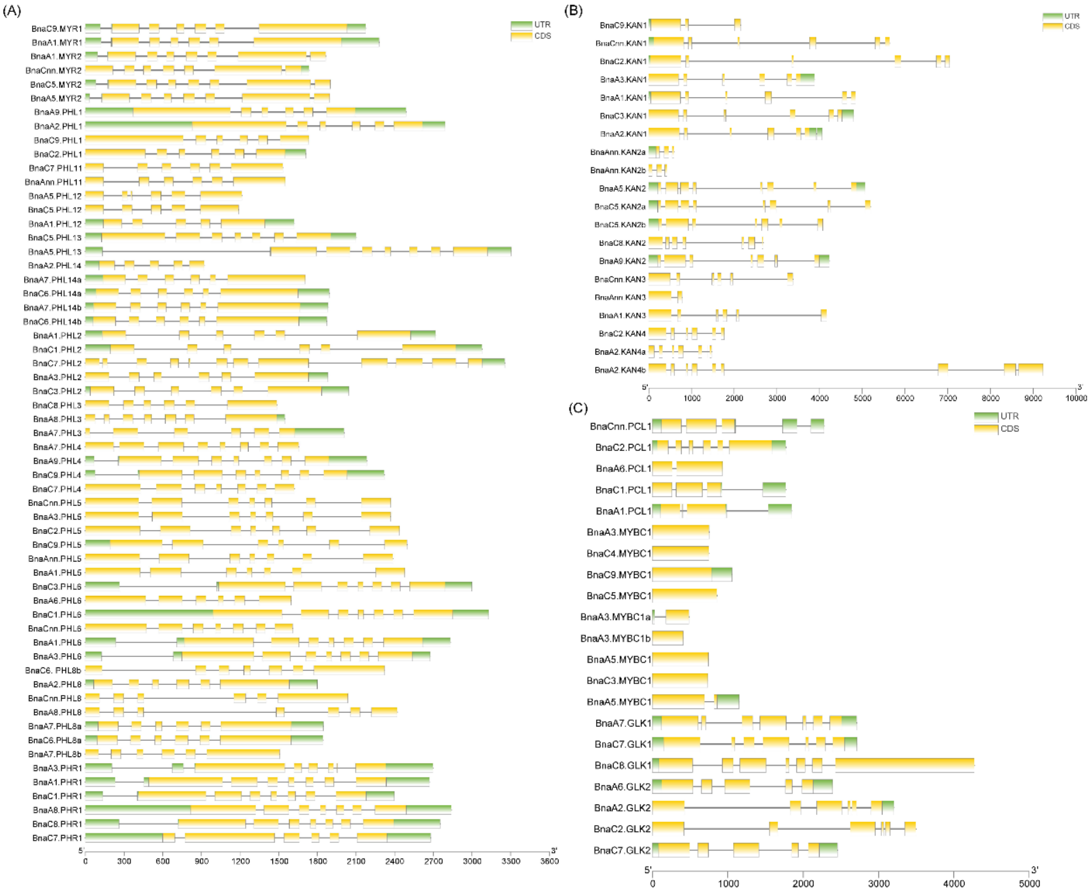

Figure S2 Exon-intron organizations of BnaGLKs (A), BnaPHLs (B), and BnaKANs (C). The green boxes represent untranslated regions, yellow boxes represent exons, and black lines represent the introns. The lengths of the exons and introns can be determined by the scale at the bottom.

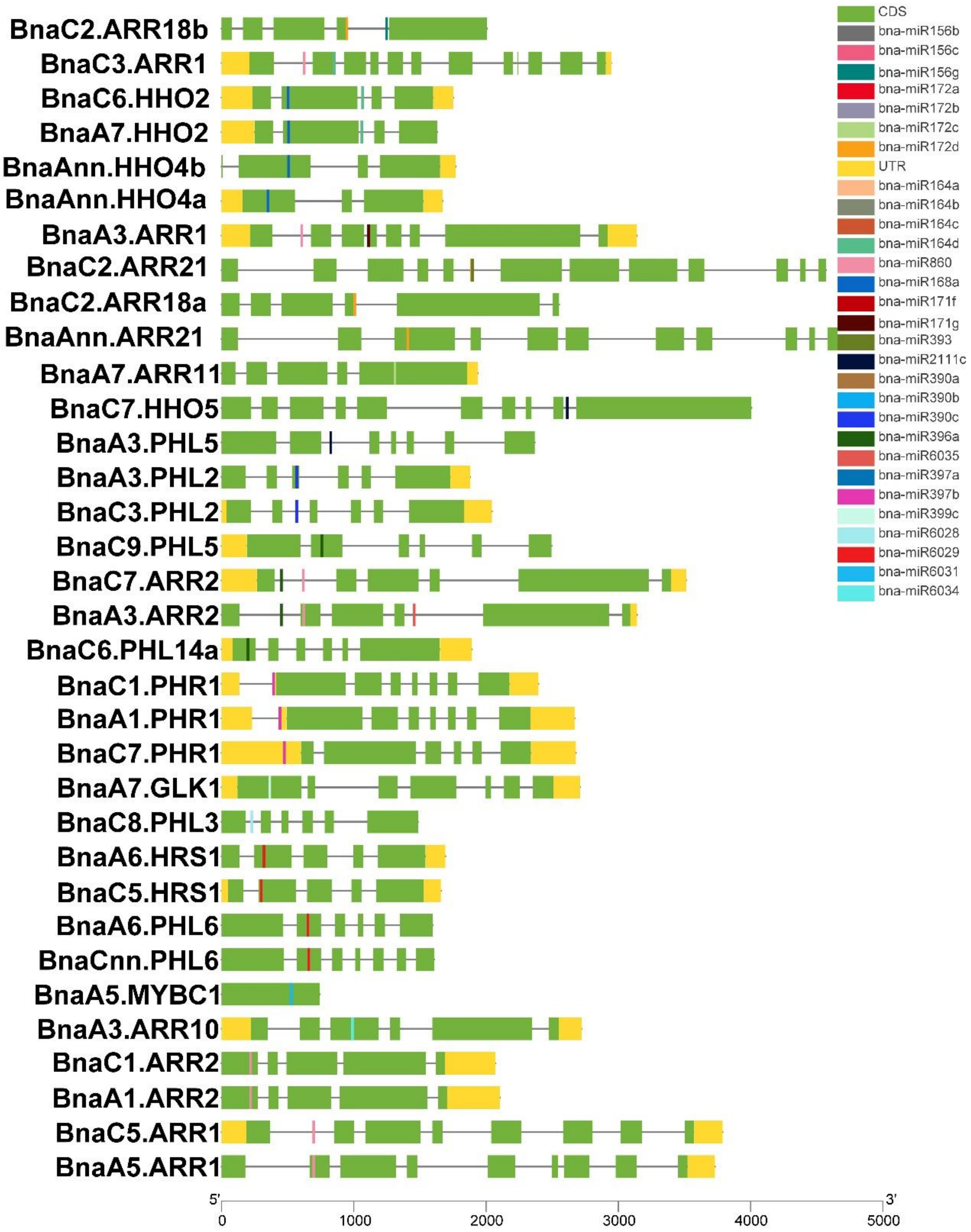

Figure S3. miRNA targeting sites of *BnaGARP* genes. Schematic illustration indicates the *BnaGARP* targeted by miRNAs.

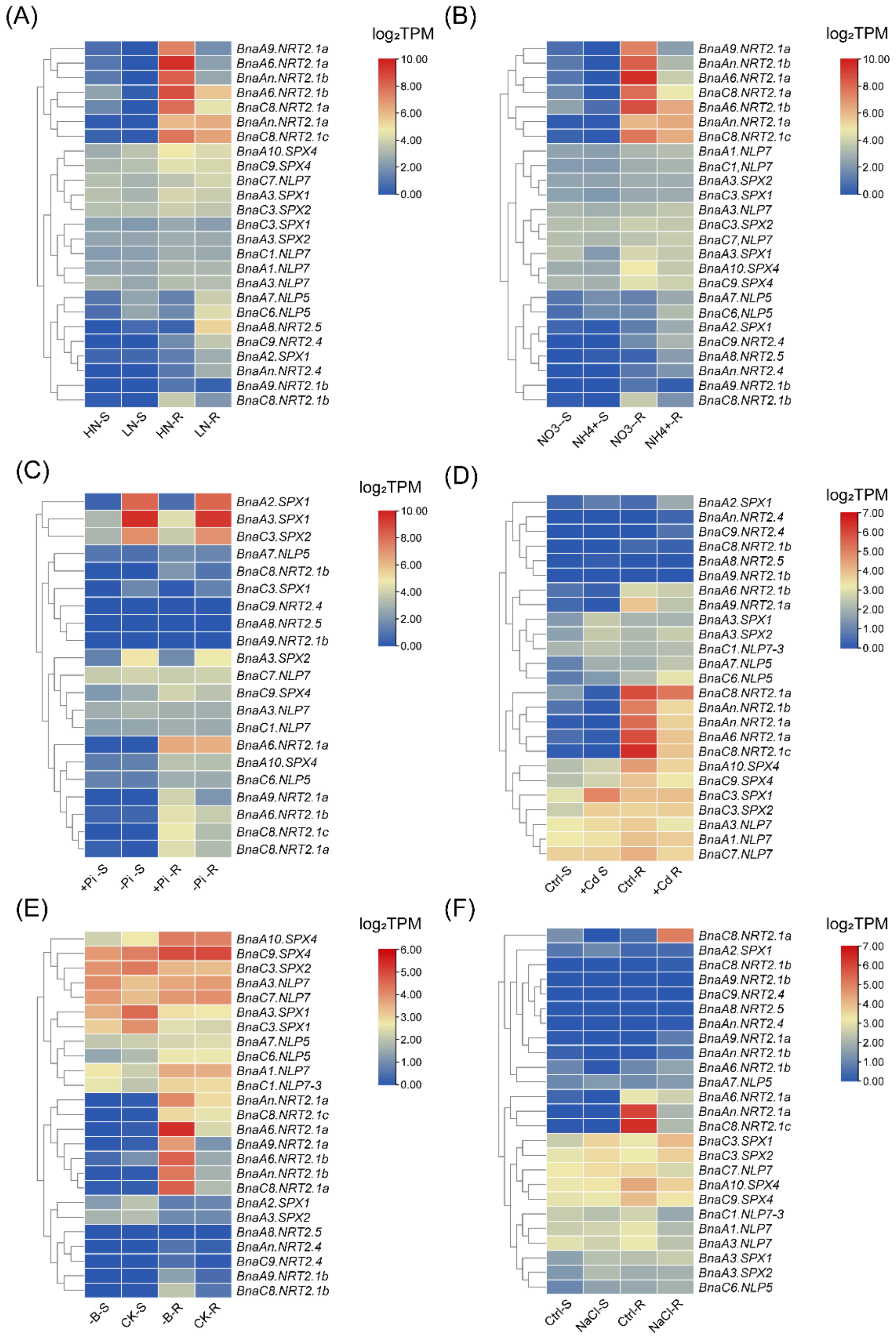

Figure S4 Expression profiles of the downstream genes that are targeted by *BnaGARP* in shoots/S and roots/R under nitrate/NO_3^- limitation (A), ammonium/NH_4^+ toxicity (B), different phosphate/Pi levels (C), cadmium/Cd toxicity (D), different boro/B levels (E), and salt stress (F) conditions. HN, high nitrate (6.0 mM NO_3^-); LN, low nitrate (6.0 mM NO_3^-). +Pi, high Pi (250 μM Pi); -Pi, low Pi (5 μM Pi). Ctrl, control (Cd-free); +Cd, Cd toxicity (10 μM $CdCl_2$). CK, high B (10 μM H_3BO_3); -B, low B (0.25 μM H_3BO_3). Ctrl, control, NaCl-free; NaCl, 200 mM (NaCl). The expression levels were normalized by log_2(TPM). TPM, transcripts per million (reads). The color scale represents relative expression levels from high (red color) to low (blue color).

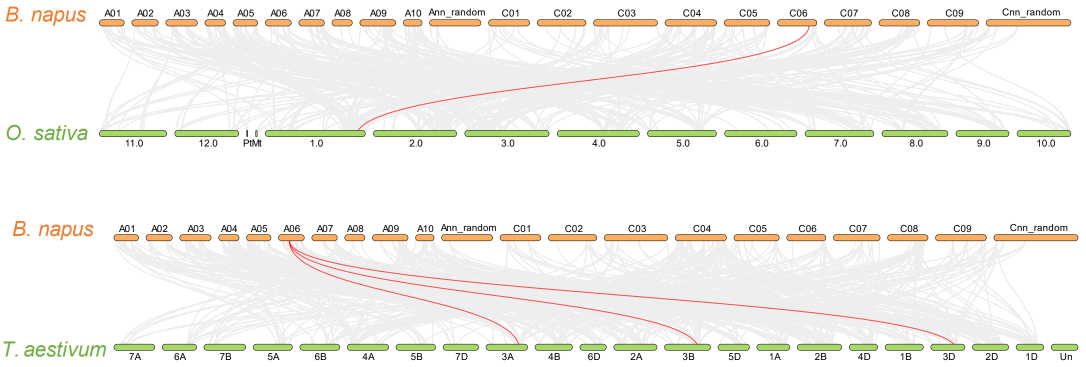

Figure S5 Synteny analysis of the *GARP* genes between *B. napus* and *O. sativa* and *T. aestivum*. Gray lines indicate all collinear blocks within *B. napus* and *O. sativa* and *T. aestivum*, while the red lines depict the orthologous relationships.
